# Clinicopathological and Immunohistochemical Characterisation of Gastric Schwannomas in 29 Cases

**DOI:** 10.1155/2014/202960

**Published:** 2014-02-11

**Authors:** Lijun Zheng, Xiaojiang Wu, Martin E. Kreis, Zhen Yu, Lijin Feng, Chunqiu Chen, Bin Xu, Zhaode Bu, Ziyu Li, Jiafu Ji

**Affiliations:** ^1^Department of General Surgery, Tenth People's Hospital of Shanghai, Tongji University, School of Medicine, Shanghai 200072, China; ^2^Department of Surgery, Beijing Cancer Hospital, Peking University School of Oncology, Beijing 100036, China; ^3^Charité University Medicine, Department of General-, Visceral- and Vascular Surgery, Campus Benjamin Franklin, Hindenburgdamm 30, 12200 Berlin, Germany; ^4^Department of Pathology, Tenth People's Hospital of Shanghai, Tongji University, School of Medicine, Shanghai 200072, China

## Abstract

Schwannomas are tumors arising from the nervous system that also occur infrequently in the gastrointestinal tract, most commonly in the stomach. This report characterizes 29 patients with benign or malignant gastric schwannomas. Surgical data and clinical follow-up information were available for 28 cases with a median postoperative duration of 57 months. Clinicopathological and immunohistochemical characteristics of benign and malignant schwannomas were analysed. Four cases (13.7%) were histologically diagnosed with malignant schwannoma. All tumors were positive for S-100 and CD56 proteins, displaying a diffuse staining pattern. Vimentin was expressed in 100% cases and all schwannomas were negative for smooth muscle actin, c-kit, and HMB-45. A significant difference was observed between the group of benign and malignant schwannomas as regards recurrences and metastasis after complete resection (*P* = 0.015). The survival time of patients with benign schwannomas was longer than the malignant group (*P* = 0.013), so gastric malignant schwannomas have a potential for recurrence and metastasis, with subsequently short survival. Complete resection with an attempt to remove all tumor tissue with negative margins is of paramount importance in the management of gastric schwannomas, particularly when they turn out to be malignant.

## 1. Introduction

Schwannomas, also known as neurinomas or neurilemmomas, are tumors of spindle cells originating from any nerve that has a Schwann cell sheath. Schwannomas of the gastrointestinal tract are very rare tumors, with the stomach being the most common location. The incidence of gastric schwannoma is reported to be about 0.2% of all gastric tumors and 4% of all benign gastric neoplasms [1]. Gastric schwannomas are thought to arise from the sheath of Auerbach's plexus or, less commonly, from Meissner's plexus [2, 3]. The tumors are generally benign, often asymptomatic, and usually are discovered incidentally at laparotomy or radiographically. The common presenting symptom is upper gastrointestinal bleeding and a palpable mass [4]. However, the tumor shared the characteristics of an exophytic mass from the gastric wall to the abdominal cavity with other gastric submucosal tumors such as gastrointestinal stromal tumors (GIST), leiomyomas, and leiomyosarcomas [5]. Therefore, the differentiation of a schwannoma from other submucosal tumors often creates preoperative diagnostic challenges. The definite diagnosis of gastric schwannoma requires histological testing to be positive for the S-100 protein, CD56, CD57, and vimentin but negative for both smooth muscle actin and c-KIT [3].

Although the majority of schwannomas are benign, a small proportion is considered malignant with a potential to local recurrence and distant metastasis. Generally gastric malignant schwannomas can be distinguished from benign schwannomas on the basis of histological and immunohistochemical features, not by clinical symptoms or imaging studies. But if on CT scan enlarged lymph nodes are seen, then this is a clear indication of the malignant potential. Preoperative differential diagnosis of gastric malignant schwannomas is generally difficult. Because of the low incidence of schwannomas and relatively limited number of cases previously reported, further analysis of larger cohorts is necessary to better understand the characterization of this particular sarcoma and diagnose it accurately. In this study, we analyzed 29 patients with benign and malignant gastric schwannomas in terms of clinicopathological and immunohistochemical parameters.

## 2. Materials and Methods

Twenty-nine schwannomas occurring in Beijing Cancer Hospital and Shanghai Tenth people's hospital were retrieved from the consultation files of the institutional archives from 1997 to 2012. We selected the cases in which the diagnosis of schwannomas was confirmed by our pathologists. Clinical data and tumor size were recorded. Paraffin blocks or recently prepared histologic sections of primary tumors were available in all cases for the following histopathologic analyses. Hematoxylin and eosin slides were reviewed in all cases. The histological features such as mitotic count per 50 fields, presence of mucosa ulcer, lymphoid reaction, architecture, and encapsulation were examined.

In our current immunohistochemical assessments, the following primary antibodies were applied with or without epitope retrieval pretreatments: anti-S-100 protein (polyclonal, 1 : 150, no pretreatment), anti-*α*-smooth muscle actin (polyclonal, 1 : 200, no pretreatment), anti-melanoma (HMB45, 1 : 50, no pretreatment), anti-CD56 (Polyclonal, 1 : 200, with pretreatment), anti-vimentin (Polyclonal, 1 : 400, with pretreatment), anti ki-67 (monoclonal, 1 : 200, with pretreatment), anti c-Kit (polyclonal, 1 : 250, with pretreatment), and anti CD34 (polyclonal, 1 : 250, with pretreatment). Heat-induced epitope retrieval in a citric acid buffer solution was used for the latter five antibodies. Immunostaining was accomplished with streptavidin biotin complex detection system and 3,3′-diaminobenzidine solution, followed by nuclear counterstaining with hematoxylin.

Clinicopathologic parameters such as age, sex, anatomic location, tumor size, mucosa ulcer, lymphoid reaction, and mitotic activity were analysed with the method of Fisher exact test to compare the clinicopathologic characteristic of benign and malignant schwannomas. The patient survival rate was displayed using the Kaplan-Meier method.

## 3. Results

### 3.1. Clinical Findings

The clinical features of the 29 patients are summarized in Table 1. There were 11 males and 18 females. Patient age ranged from 43 to 81 years (mean 63.5, median 66). 20 patients had complaints before they went to see the doctor. The main complaint was gastric pain (including abdominal pain), which was seen in 6 (20.7%) patients. Other complaints included gastrointestinal bleeding which was seen in 4 (13.8%) cases (usually occult blood in stool and melena), epigastric pain or discomfort which was seen in 2 patients, gastric discomfort which was seen in 5 cases (including fullness of stomach), poor appetite (*n* = 1), and weight loss (*n* = 1). The tumor in one patient was detected during CT examination for unknown symptoms. Nine cases were incidental findings during surgeries or examinations for unrelated diseases. The tumors in 6 patients were discovered incidentally during cholecystectomy (*n* = 3), laparoscopic appendectomy (*n* = 2), or colectomy for colon cancer (*n* = 1). Two cases were seen at the time of abdominal ultrasound for cholecystolithiasis. One gastric schwannoma was detected during CT as part of follow-up examination after surgery for lung cancer. None of the patients has history of neurofibromatosis type 1 (NF1) syndrome or neurofibromatosis type 2 (NF2) syndrome.

All patients were treated by surgery including partial gastrectomy (*n* = 13), gastric wedge resection (*n* = 8), subtotal gastrectomy (*n* = 7), and total gastrectomy (*n* = 1). The status at surgery and clinical follow-up information were available in 28 cases with a mean postoperative duration of 57 months (range: 6 to 157 months). The tumor locally recurred in one patient and liver metastases were present after surgery in another case. These two patients were diagnosed with malignant gastric schwannoma after operation and died of it. Our followup revealed 19 patients to be alive without recurrences for 6 to 157 months (mean 53 months, median 47 months). Seven patients had died of unrelated causes which included cardiocerebral vascular accidents (*n* = 3), traffic accident (*n* = 1), lung cancer (*n* = 1), acute pancreatitis (*n* = 1) and diabetic kidney (*n* = 1), during 25 to 132 months (mean 74 months, median 80 months). One patient was lost to followup.

### 3.2. Pathologic Findings

**Figure 1:**
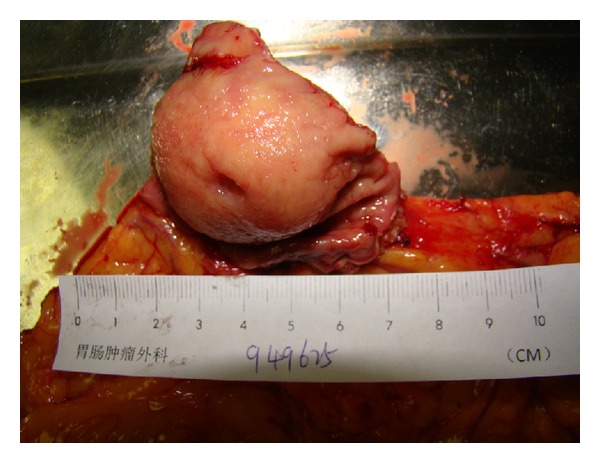
Gross appearance of resected specimen of one case. The gastric submucosal schwannoma encapsulated with normal mucosa, with ulceration on the top.

The most common origin was gastric antrum (*n* = 10, 34.5%), followed by greater curvature (*n* = 7, 24.1%), gastric fundus (*n* = 6, 20.7%), lesser curvature (*n* = 5, 17.2%), and cardia (*n* = 1, 3.4%). The tumors range varied in greatest diameter from 2.1 cm to 7.9 cm, with a mean of 5.06 ± 1.38 cm (median 5.2 cm). In nine cases, the tumors showed expansive growth with distinct margin, encapsulated by a thin membrane (Figure 1). On section, the tumors were described as white or yellow-white with shiny, whorled texture. In 16 cases the tumors involve the layer of submucosa. Muscularis propria was involved in 10 patients. The tumors invaded the layer of submucosa and muscularis propria with gross mucosal ulceration in 3 patients.

Under microscopic examination, an ulcer of mucosa was present in most tumors (about 82.8%) and no definite true capsules were histologically detected in any cases. The tumors were composed entirely or mainly of short spindle shaped cells with no nuclear atypia in 21 cases. Spindle cells of neoplasm were described in a palisade manner in 19 patients. In 15 tumors, the tumors show a lymphocytic cuffing at the peripheral part. Diffuse intratumoral lymphoid infiltration was seen in all cases, with plasma cells present in 12. The tumor cells were mostly uniform, and had palisading nuclear. The median mitotic rate was 2 mitoses/50 high-power fields (HPFs) (range: 0 to 20/50 HPFs, mean: 4.3/50 HPFs). Four tumors had 15–20 mitoses per 50 HPFs with high degree of nuclear atypia. Based on the presence of mitotic figures and nuclear atypia, four cases (13.7%) were diagnosed with malignant schwannoma. But without pathologic data were found about lymph nodes involved around specimen in malignant cases.

### 3.3. Immunohistochemical Findings

**Figure 2:**
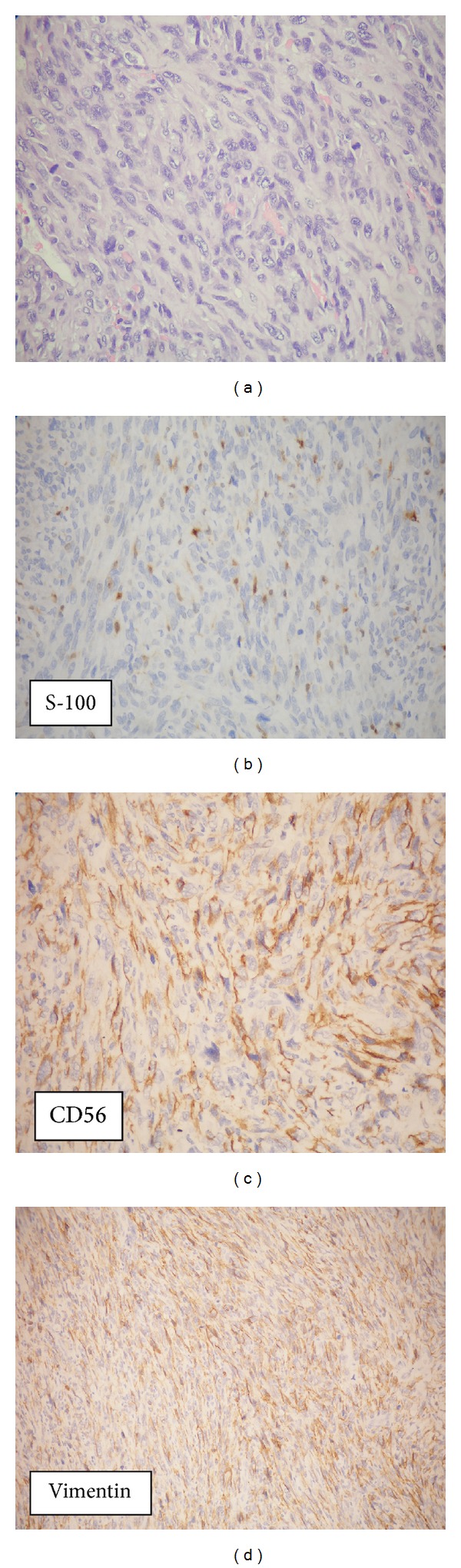
One malignant gastric schwannoma case. (a) Tumor cells show high variation of nuclear shape and size with mitosis (H&E, original magnification ×200). Diffuse positive staining is seen for (b) S-100 protein (positive < 20%), (c) CD56 (>50%), and (d) vimentin (>50%) in tumor cells.

The immunohistochemical results are summarized in Table 2. All tumors were positive for S-100 and CD56 protein, displaying a diffuse staining pattern. Immunoreactivity for Ki-67 was seen focally in all but one case. Vimentin was 100% expressed in all cases tested (Figure 2). Proteins of CD34 were focally expressed in minor fractions of the tumor vessels (13.8%). All examined 29 cases were negative for smooth muscle actin, c-kit, and HMB-45.

### 3.4. Statistical Analysis

**Figure 3:**
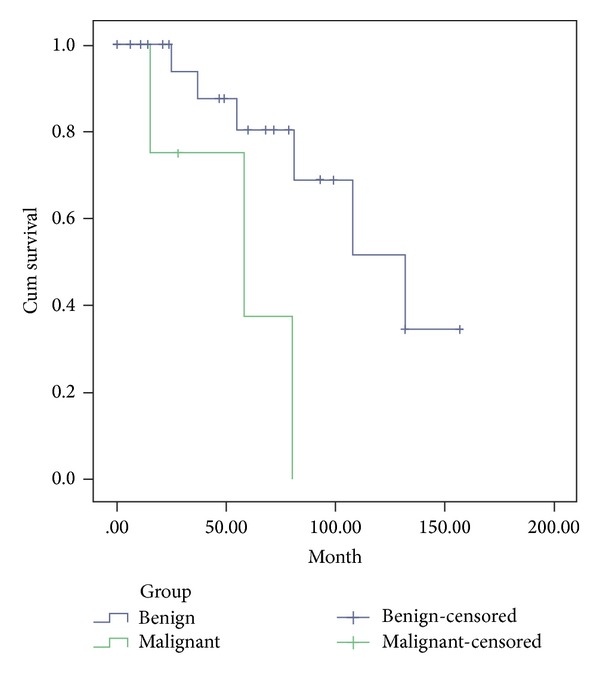
Cumulative survival of patients for the group of benign schwannoma and malignant schwannoma. (*P* = 0.013) for comparison between two groups.

As is shown in Table 3, the clinical and pathologic variables of benign and malignant schwannomas were compared to access the potential diagnosis value from their clinicopathologic characteristics. No significant diagnosis value was identified according to gender, age (< versus ≥ median age), anatomic location, tumor size (< versus ≥ median size), and ulcer of mucosa. Recurrence was observed in one of 4 malignant cases (25.0%) and one case (25.0%) developed liver metastasis after surgery. There was a difference between the group of benign and malignant schwannomas in recurrence and metastatic rate after complete resection (*P* = 0.015). Median disease-free survival time of the benign schwannomas group was 132.0 months with no observed recurrence, while it was 58 months in the group of malignant schwannomas (*P* = 0.013) (Figure 3).

## 4. Discussion

Schwannomas are slowly-growing encapsulated tumors composed of Schwann cells in a collagenous matrix. As the tumor enlarges, it displaces the nerve to the periphery of the tumor, preserving neural function [6]. According to previous reports, gastric schwannomas frequently occur in female individuals between the ages of 50 to 60 years and are usually solitary lesions arising from the lesser curvature of the stomach [7–9]. In our series of patients, one-third of the gastric schwannomas were located in gastric antrum, with one-quarter in greater curvature of gastric body and gastric fundus, respectively. A female predominance was also observed in our study with a ratio of almost 2 : 1 (female to male) [10]. Patients' age with gastric schwannomas was around 60 years. They are often asymptomatic or with mild abdominal discomfort discovered incidentally at laparotomy or radiographically. Only bleeding may be present in the case of deep ulceration [11], and a mass may be palpated in the epigastric area when exophytic growth has occurred. Most patients in our series were suffering from abdominal pain, gastrointestinal bleeding, and epigastric pain or discomfort. Our results were consistent with Burneton's review that most patients presented with bleeding, followed by abdominal pain [12].

The most frequently ordered diagnostic test in our series was upper-GI endoscopy performed before surgical exploration. Endoscopy may identify submucosal lesions, with or without ulceration. Recently, endoscopic ultrasonography is evaluated in the diagnosis of gastric schwannomas [13]. But in our series, endoscopic ultrasound added little information for planning extent and type of the operation. This may be due to the fact that the lesions of gastric schwannomas are usually encased by intact mucosa and predominantly involve the submucosa and muscularis propria. Computed tomography (CT) scans are helpful in determining the tumor size and its relation to the adjacent organs during evaluation before surgery. However, when the tumor has the characteristics of an exophytic mass from the gastric wall to the abdominal cavity, the differentiation of a schwannoma from other submucosal tumors is very difficult on preoperative examination. Thus, although it may be helpful to gain limited information through GI endoscopy, CT, sonography, PET, and MRI, the definitive diagnosis of gastric schwannomas is determined by pathological examination [2]. The histological origin of submucosal tumors of the gastrointestinal tract has only recently been clarified by immunohistological procedures. Strong positivity for S-100 protein and negative staining for CD117 (c-kit) characterize such tumors [3].

Laparoscopic gastric resection, including wedge resection, subtotal resection, or near-total resection, is the treatment of choice for gastric schwannoma. Complete resection of the tumor is of paramount importance [14]. Like other soft tissue sarcomas, gastric schwannomas rarely metastasize to lymph nodes, and thus surgical lymphadenectomy for gastric schwannomas is not routinely performed. So, no data were found about lymph nodes involved around specimen in our cases. The postoperative prognosis for solitary benign schwannoma is excellent. The patients with benign gastric schwannoma survived without recurrence and metastasis in our study during followup. Recurrent disease is generally associated with an incomplete surgical margin in malignant cases. It is of note that gastric schwannoma's malignant potential should be determined prior to surgery. However, distinction between benign and malignant gastric schwannomas is mainly based on immunohistochemical features and therefore identified difficultly by clinical tests and imaging. In fact, preoperative differential diagnosis of gastric submucosal tumor is generally difficult. So the treatment strategy for gastric schwannoma is complete resection with negative margins and avoiding rupture of the tumors regardless of whether the tumor is malignant or benign [15]. Molecular and target therapy as great advances plays an important role in the treatment of gastrointestinal mesenchymal tumors. Altuna et al. [16] assumed that tyrosinase inhibitor such as imatinib may have therapeutic effect in treating vestibular schwannoma with high expression rate of platelet-derived growth factor receptor (PDGFR) and c-kit. However, the efficiency of chemotherapy remains uncertain.

Malignant schwannoma (malignant peripheral nerve sheath tumor, MPNST) is a high grade sarcoma with a potential for local recurrence and distant metastasis that may occur at any site in the body where there is neural tissue. The histological criteria for the diagnosis of a malignant schwannoma are based on the presence of mitotic figures, nuclear atypia, part of necrosis, and other pathological features, not on clinical symptoms or imaging studies [17]. As a fact, in this report, criteria aforementioned to classify the tumors as benign or malignant were applied. Mitoses of the tumor over 15/50 HPFs and high degree of nuclear atypia with lower S-100 and higher Ki-67 expression were considered the characteristic of malignant schwannoma. The incidence of malignant gastric schwannoma was reported to be about 10–15% of gastric schwannomas [15]. The prognosis for malignant schwannoma patients is poor and is characterized by rapidly progressive disease and poor response to chemotherapy. Most patients die before 2 years, with an average 5-year survival of 23% [18]. In our study, two patients with malignant schwannoma occurred and developed recurrence and metastasis after operation and died from it. As a logical consequence, median disease-free survival time of the malignant schwannomas group was a lot shorter than that of the benign group. Furthermore, results also may be confirmed that the mitotic count and nuclear atypia were highly related to the prognosis, but further patients with malignant schwannomas should be analysed during future research.

## 5. Conclusion

Our experience confirmed that infrequently diagnosed gastric schwannomas have a female predominance. The clinic course of gastric schwannomas are usually benign, slow-growing, and asymptomatic. Gastric malignant schwannomas are extremely rare, reported with a high potential for recurrence and metastasis in our series. Histological and immunohistochemical features are of paramount importance to differentiate between benign gastric schwannoma, malignant gastric schwannoma, and other spindle cell sarcomas. Finally, complete resection with an attempt to remove all gross disease and achieving negative margin is a fundamental surgical principle in the management of gastric schwannomas. However, further research is necessary to better understand features of malignant schwannomas.

## Figures and Tables

**Table 1:** Clinicopathologic features of 29 cases of gastric schwannomas.

Case	Age (y)/sex	Clinical presentation	Site	Size (cm)	Mitoses/50 HPFs	Outcome	Follow-up time (mo)
1	54/F	Incidental	Gastric antrum	3.5	0	ANED	157
2	78/M	GI bleeding	Gastric fundus	6.5	2	DUC	132
3	81/F	Epigastric pain and mass palpated on a doctor's visit for another disease	Gastric body (lesser curvature)	7.0	1	DUC	108
4	43/F	Gastric pain	Gastric antrum	4.0	6	ANED	72
5	54/F	Incidental	Gastric fundus	5.3	4	ANED	47
6	67/F	Incidental	Gastric antrum	3.2	1	ANED	23
7	78/M	Poor appetite	Gastric antrum	2.6	0	DUC	37
8	73/F	Gastric distress	Gastric body (greater curvature)	4.4	0	ANED	11
9	54/F	Incidental	Gastric antrum	2.9	2	DUC	25
10	46/F	Gastric pain	Gastric body (greater curvature)	5.2	6	ANED	24
11	76/F	Weight loss	Gastric antrum	2.1	1	ANED	21
12	64/M	Incidental on CT for lung cancer followup	Gastric fundus	7.5	9	ANED	14
13	57/M	Detected during abdominal sonography for cholecystolithiasis	Gastric fundus	4.3	2	ANED	14
14	45/M	Incidental	Gastric body (lesser curvature)	5.5	1	ANED	10
15	66/F	GI bleeding	Gastric body (lesser curvature)	4.0	0	LTF	0
16	79/M	Intermittent gastric discomfort	Gastric antrum	3.0	0	ANED	6
17	52/M	Detected during abdominal sonography for cholecystolithiasis	Gastric antrum	6.5	5	ANED	132
18	67/M	Gastric pain	Gastric body (greater curvature)	6.7	15	DUC	80
19	73/F	Hematemesis, melena	Gastric antrum	5.2	20	DMR	15
20	61/M	Gastric pain	Gastric antrum	5.7	16	ANED	28
21	55/F	Nausea and gastric discomfort	Gastric body (lesser curvature)	3.8	1	ANED	93
22	67/F	Nausea and occult blood in stool	Gastric fundus	7.9	3	DUC	81
23	60/M	Incidental	Gastric fundus	5.7	2	ANED	49
24	66/F	Fullness of stomach	Gastric body (greater curvature)	6.9	5	ANED	60
25	65/F	Detected during CT for unknown symptoms	Gastric body (greater curvature)	6.2	19	DMR	58
26	43/F	Epigastric discomfort	cardia	3.8	3	ANED	99
27	78/F	Fullness of stomach	Gastric body (greater curvature)	4.2	1	ANED	68
28	69/M	Abdominal pain	Gastric body (greater curvature)	6.0	0	DUC	55
29	70/F	Gastric pain	Gastric body (lesser curvature)	7.2	1	ANED	79

DUC: died of unrelated causes; ANED: alive with no evidence of disease; LF: lost to followup; DMR: died of metastasis or recurrent of the primary disease.

**Table 2:** Summarized immunohistochemical results of gastric schwannomas.

Antigen (antibody)	No. cases examined	No. positive cases (%)	No. cases with ranges of positive tumor cells		
			<20%	20% to 50%	>50%
S-100 protein	29	29	2	2	25
HMB45	29	0	0	0	0
CD56	29	29	2	8	19
CD34	29	4	3	1	0
*α*-Smooth muscle actin	29	0	0	0	0
c-Kit	29	0	0	0	0
Ki-67	29	21	5	13	3
Vimentin	27	27	6	9	12

**Table 3:** Comparison of clinical and pathologic variables between benign and malignant gastric schwannoma.

Variables	Benign schwannoma (*n* = 25)	Malignant schwannoma (*n* = 4)	*P* value
Age			
<median age	12	2	1.00
≥median age	13	2	
Gender			
Male	9	2	0.622
Female	16	2	
Anatomic location			
Gastric antrum	8	2	0.299
Gastric fundus	6	0	
Gastric body (lesser curvature)	5	0	
Gastric body (greater curvature)	5	2	
Cardia	1	0	
Tumor size			
<median size	13	0	0.107
≥median size	12	4	
Ulcer of mucosa	21	3	0.553
Recurrences or metastatic	0	2	0.015
Survival status			
Alive with no evidence of disease	18	1	0.021
Died of unrelated causes	6	1	
Died of metastasis or recurrent of the primary disease	0	2	
Lost to followup	1	0	

## References

[1] Lin C-S, Hsu H-S, Tsai C-H, Li W-Y, Huang M-H (2004). Gastric schwannoma. *Journal of the Chinese Medical Association*.

[2] Fujiwara S, Nakajima K, Nishida T (2013). Gastric schwannomas revisited: has precise preoperative diagnosis become feasible?. *Gastric Cancer*.

[3] Daimaru Y, Kido H, Hashimoto H, Enjoji M (1988). Benign schwannoma of the gastrointestinal tract: a clinicopathologic and immunohistochemical study. *Human Pathology*.

[4] Melvin WS, Wilkinson MG (1993). Gastric schwannoma: clinical and pathologic considerations. *The American Surgeon*.

[5] Miettinen M, Majidi M, Lasota J (2002). Pathology and diagnostic criteria of gastrointestinal stromal tumors (GISTs): a review. *European Journal of Cancer*.

[6] Hunter VP, Burke TW, Crooks LA (1988). Retroperitoneal nerve sheath tumors: an unusual cause of pelvic mass. *Obstetrics and Gynecology*.

[7] Tahir TM, Anwar S, Naseem N, Mansoor-Ul-Haq H, Saqib M (2010). Gastric schwannoma in a female patient with pulmonary tuberculosis—a clinicopathological assessment and diagnosis. *Malaysian Journal of Medical Sciences*.

[8] Yoon W, Paulson K, Mazzara P, Nagori S, Barawi M, Berri R (2012). Gastric schwannoma: a rare but important differential diagnosis of a gastric submucosal mass. *Case Reports in Surgery*.

[9] Shelat VG, Li K, Naik S (2013). Abdominal schwannomas: case report with literature review. *International Surgery*.

[10] Voltaggio L, Murray R, Lasota J, Miettinen M (2012). Gastric schwannoma: a clinicopathologic study of 51 cases and critical review of the literature. *Human Pathology*.

[11] Williamson JML, Wadley MS, Shepherd NA, Dwerryhouse S (2012). Gastric schwannoma: a benign tumour often mistaken clinically, radiologically and histopathologically for a gastrointestinal stromal tumour—a case series. *Annals of the Royal College of Surgeons of England*.

[12] Bruneton JN, Drouillard J, Roux P (1983). Neurogenic tumors of the stomach. Report of 18 cases and review of the literature. *Fortschritte auf den Gebiete der Rontgenstrahlen und der Nuklearmedizin*.

[13] Lok K-H, Lai L, Yiu H-L, Szeto M-L, Leung S-K (2009). Endosonographic surveillance of small gastrointestinal tumors originating from muscularis propria. *Journal of Gastrointestinal and Liver Diseases*.

[14] Euanorasetr C, Suwanthanma W (2011). Gastric schwannoma presenting with perforation and abscess formation: a case report and literature review. *Journal of the Medical Association of Thailand*.

[15] Takemura M, Yoshida K, Takii M, Sakurai K, Kanazawa A (2012). Gastric malignant schwannoma presenting with upper gastrointestinal bleeding: a case report. *Journal of Medical Case Reports*.

[16] Altuna X, Lopez JP, Yu MA (2011). Potential Role of imatinib mesylate (Gleevec, STI-571) in the treatment of vestibular schwannoma. *Otology and Neurotology*.

[17] Loffeld RJ, Balk TG, Oomen JL, van der Putten AB (1998). Upper gastrointestinal bleeding due to a malignant Schwannoma of the stomach. *European Journal of Gastroenterology and Hepatology*.

[18] Bees NR, Ng CS, Dicks-Mireaux C, Kiely EM (1997). Gastric malignant schwannoma in a child. *British Journal of Radiology*.

